# Antimicrobial and Immunomodulatory Activity of Herb Extracts Used in Burn Wound Healing: “San Huang Powder”

**DOI:** 10.1155/2021/2900060

**Published:** 2021-10-12

**Authors:** Jia-Ru Wu, Yu-Chu Lu, Sung-Jen Hung, Jung-Hsing Lin, Kai-Chih Chang, Jhong-Kuei Chen, Wan-Ting Tsai, Tsung-Jung Ho, Hao-Ping Chen

**Affiliations:** ^1^Integration Center of Traditional Chinese and Modern Medicine, Hualien Tzu Chi Hospital, Hualien 97002, Taiwan; ^2^Department of Chinese Medicine, Hualien Tzu Chi Hospital, Hualien 97002, Taiwan; ^3^Department of Dermatology, Hualien Tzu Chi Hospital, Hualien 97002, Taiwan; ^4^Department of Laboratory of Medicine and Biotechnology, Tzu Chi University, Hualien 97004, Taiwan; ^5^School of Post-Baccalaureate Chinese Medicine, Tzu Chi University, Hualien 97004, Taiwan; ^6^Department of Biochemistry, Tzu Chi University, Hualien 97004, Taiwan

## Abstract

“San Huang Powder,” a nonsterile milled herb powder, is frequently used to treat burn wounds in traditional Chinese herbal medicine. However, treating a wound with a nonsterile dressing or reagent is not compatible with the current guidelines in modern medicine. Therefore, we investigated the bactericidal and anti-inflammatory activities of four herb extracts used in “San Huang Powder” *in vitro*. Meanwhile, an *in vivo* porcine model with superficial second-degree burns was used for the experiments since the size and skin composition of pigs are the closest to that of the human body. The minimal bactericidal concentration (MBC) of the herb extracts was determined. The *in vitro* assay indicated that Rhubarb and Phellodendron bark extracts decreased the levels of inflammatory cytokines, IL-8, and GM-CSF on LPS-induced HMEC-1 cells. In accordance with this result, the histopathological evaluation results showed that the efficacy of “San Huang Powder” containing both herb materials was much better than the group without Rhubarb. Our results not only provide a basis to understand why “San Huang Powder” has been used to clinically treat wounds without sterilization directly since ancient times but also show the advantages of using multiple herb materials simultaneously on wound sites to prevent infection during treatment. Rhubarb is the recommended ingredient involved in the preparation of “San Huang Powder” to ensure the healing efficacy of burn wounds.

## 1. Introduction

“San Huang Powder” is a widely used traditional Chinese herbal medicine. The name of this preparation was first mentioned in an ancient medical book, Beiji Qianjin Yao Fang, published in 652 B.C. It is well known for the treatment of first- and second-degree burn wounds [1–3]. Literally, “San Huang” means that this medicine is made from three different yellow-colored herbs. However, the recipes of “San Huang Powder” vary in different ancient medical books. Rhubarb, Scutellaria root, Phellodendron bark, and Coptidis rhizome are the four most frequently used materials to prepare “San Huang Powder.” Moreover, Rhubarb's efficacy in treating burn wounds was recently reported [4, 5]. However, there is no systematic study comparing the efficacy of different “San Huang Powder” recipes for the treatment of burn wounds to date.

The milled herbal powder without sterilization is used to treat wounds directly in folk medicine. However, modern medicine physicians constantly criticize this treatment due to concerns relating to infection control. In this study, an *in vitro* bactericidal activity assay was performed to provide more clarity on this issue. The *in vitro* immunomodulatory activity of herbal materials was also investigated. Since pig skin structure and function have the closest resemblance to that of humans [6, 7], we used an *in vivo* porcine burn model in healing burn wounds to examine the efficacy of two different “San Huang Powder” recipes. The results obtained in this study provide the scientific basis for its clinical use and insight for preparation of next generation “San Huang Powder” extract.

## 2. Materials and Methods

The Institutional Animal Care and Use Committee, National Laboratory Animal Center, Taiwan, ROC, approved all experimental animal procedures (Permission number: NLAC (TN)-107-M-009R1).

### 2.1. Materials

Berberine hydrochloride was purchased from TCI Co., Ltd. (Tokyo, Japan). Chrysin was obtained from Acros Organics (Geel, Belgium). Chrysophanol was purchased from Sigma-Aldrich (St. Louis, MI, USA). Vascular endothelial growth factor (VEGF) was bought from B & D Systems (Minneapolis, MN, USA). Rhubarb (dried stem and root from *Rheum palmatum* LINN) was a product of Da Rong Co., Ltd. (Tao Yuan City, Taiwan) (batch number: DK1070932). Scutellaria root (dried root from *Scutellaria baicalensis Georgi*) was the product of He Kang Chinese Medicine Co., Ltd. (New Taipei City, Taiwan) (batch number: 0704). Phellodendron bark (dried bark from *Phellodendron amurense* Ruprecht) was the product of Jin Rong Co., Ltd. (New Taipei City, Taiwan) (batch number: AG80303). Coptidis rhizome (dried rhizome of *Coptis chinensis* Franch) was the product of Fu Ji Co., Ltd. (Kaohsiung City, Taiwan) (batch number: FG0013). Further identification analysis of these plant-based materials was done by HPLC, as described below. The raw herb materials were ground into fine powder using a coffee grinder (Model: ECG3003S, Electrolux, New Taipei City, Taiwan). The milled powder was further sieved using ultrafine 100 mesh stainless steel filter and stored in a dry cabinet. The recipes of two different types of “San Huang Powder” are listed in Table 1.

### 2.2. Determination of Reference Standard Content in Rhubarb, Scutellaria Root, Phellodendron Bark, and Coptidis Rhizome by HPLC

All experiments were performed using a Hitachi L-7000 HPLC system (Hitachi, Ltd., Tokyo, Japan), equipped with a L-7100 quaternary gradient pump and a L-7450 photo diode array detector. Hitachi HSM software was used for machine control and data collection and processing. The analytical column used was the *μ*Bondapak™ C18 Column, 125 Å, 10 *μ*m, 3.9 × 300 mm (Waters Corporation, Milford, Massachusetts, USA).

A 1 g sample of each dried herb material was ground into fine powder, using a coffee grinder (Model: ECG3003S, Electrolux, New Taipei City, Taiwan). The herbs were then extracted twice with the following solvents: Rhubarb: 10 mL methanol, Scutellaria root: 10 mL of ethanol, Phellodendron bark: 10 mL of methanol, and Coptidis rhizome: 10 mL of 70% ethanol. One gram of grounded solids were weighed and dissolved in 10 ml of solvent. After ultrasonicating for 30 minutes at 25°C, extracts were transferred to a new glass vial using disposable glass Pasteur pipettes. Another 10 ml of solvent was then added and ultrasonicated for another 30 minutes at 25°C. Undissolved particles were removed by centrifugation at 2500 ×g for 10 minutes at 25°C and filtered through a 0.22 *μ*m syringe filter. The final volume of the extract was adjusted to 20 ml. To calculate the extraction yield (mass of extract/mass of dry matter), 1 ml extracts were dried under vacuum at 25°C overnight in a Savant SpeedVac Vacuum Concentrator (Thermo Fisher Scientific Inc., Waltham, Massachusetts, USA). Folin-Ciocalteu method was used to determine the total phenolic content in the extracts [8]. Total phenolic content of the extract samples was expressed as gallic acid equivalent (GAE) milligrams per gram of the extract.

The methanol extract of Rhubarb was separated using a gradient elution of solvent A (10% CH_3_CN, containing 0.1% H_3_PO_4_) and solvent B (90% CH_3_CN, containing 0.1% H_3_PO_4_) at a flow rate of 1 ml/min [9]. The UV detection wavelength was 254 nm. The ethanol extract of Scutellaria root was separated using a gradient elution of solvent A (10% CH_3_CN, containing 0.1% H_3_PO_4_) and solvent B (90% CH_3_CN, containing 0.1% H_3_PO_4_) at a flow rate of 1 ml/min. The UV detection wavelength was 280 nm. The methanol extracts of Phellodendron bark were separated using a gradient elution of solvents A (10% CH_3_CN, containing 0.1% H_3_PO_4_) and B (90% CH_3_CN, containing 0.1% H_3_PO_4_) at a flow rate of 1 ml/min. The UV detection wavelength was 260 nm. The ethanol extract of Coptidis rhizome was separated using a gradient elution of solvents A (0.1% KH_2_PO_4_ buffer) and B (100% CH_3_CN) at a flow rate of 1 ml/min [10]. The UV detection wavelength was 260 nm. The HPLC elution programs for the four herbs are presented in Table 2.

### 2.3. Determination of the Minimal Bactericidal Concentration (MBC) of the Herbal Materials

The minimal bactericidal concentration (MBC) of the herbal materials used in this study to kill the following, *Acinetobacter baumannii* Bouvet and Grimont (ATCC® 19606™), *Acinetobacter baumannii* Bouvet and Grimont (ATCC® 17978™), *Elizabethkingia meningoseptica* BCRC 10677, *Escherichia coli* DH5*α*, *Pseudomonas aeruginosa* PAO1, *Propionibacterium acnes* PS023, *Staphylococcus epidermidis* TCU-1 BCRC 81267, and *Staphylococcus aureus* subsp. aureus TCU-2 BCRC 81268, was determined. *P. acnes* PS023 is an erythromycin- and clindamycin-resistant clinical isolate. One gram of each herb material was extracted with 4 mL methanol at room temperature, overnight. The herbal methanol extracts (500 *µ*L each) were dried using a Savant SpeedVac Vacuum Concentrator and dissolved in 25 *µ*L DMSO. The DMSO stock of each herb extract was further diluted (10x) with the Rein-forced Clostridial Medium for *P. acnes* PS023 and Mueller Hinton broth for the other bacteria. The adjusted herb extract was serially diluted into multiple wells on a 96-well plate to obtain a gradient. After overnight growth at 37°C, the wells which were clear were evaluated for colony-forming units per mL (CFU/mL) on agar plates. Only *P. acnes* PS023 grow in anaerobic chamber using a mixture of 10% CO_2_, 10% H_2_, and 80% N_2_.

### 2.4. Animal Experiments

**Figure 1:**
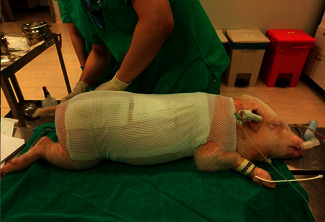
Burn wounds were covered with sterile gauze dressing after treatment with CA, and TA.

Three female Lee-Sung pigs were purchased from the Department of Animal Science and Technology, National Taiwan University, Taiwan. The pigs were 4.5 months old (average weight: 18.0 kg) and had similar body shapes. Each animal was maintained as described previously [7]. The Institutional Animal Care and Use Committee, National Laboratory Animal Center, Taiwan, ROC, approved all experimental animal procedures (Permission number: NLAC(TN)-107-M-009R1). General anesthesia was maintained by isoflurane via inhalation. Intramuscular Zoletil (5 mg/kg) and Xylazine (2.2 mg/kg) and subcutaneous Atropine (0.05 mg/kg) were used for sedation. Subcutaneous injection of Buprenorphine (0.05 mg/kg) for pain relief and oral administration of Enrofloxacin (5 mg/kg) were performed for infection control during the operation. Meanwhile, isoflurane inhalation and Lidocaine (2%) spray anesthesia were administered (Figure 1). A second-degree burn wound was made with the help of a burn device (Model YLS-5Q, Yi Yan Tech. Co., Ltd., Shandong, China), consisting of a 4 cm diameter heating probe. The setting of the burn device for contact pressure, temperature, and time was 500 g, at 90°C for 20 sec, respectively. The procedure was performed under aseptic conditions to create uniform dermal scalding wounds on the three adult minimum disease female Lee-Sung pigs. The burn wounds were created on six different locations on the dorsal side of each animal. Sterile gauze was used to keep the wounds clean. Two different “San Huang Powder” compositions, test article (TA) and control (CA), were directly applied to the wound surface, and dressings were replaced daily (Table 3). In traditional Chinese medicine, the physician directly sprays “San Huang Powder” over the wound site. About one gram of “San Huang Powder” was used over the wound area (12 cm^2^). Oral cephalexin (20 mg/kg) and meloxicam (0.4 mg/kg) were administered twice daily during the first seven days. No apparent abnormalities were seen during the experimental process until the sacrifice. Euthanasia of all animals was performed one day after completion of the study by using pentobarbital (120 mg/kg). Once euthanasia was performed, the collection of tissues was initiated immediately.

The wound sites were photographed on days 7, 14, and 21 after the burn injury. The dermal wound tissue was sampled using a 6 mm biopsy punch (Lot no.: 17L13, Integra LifeSciences, Plainsboro, NJ, USA) and preserved in 10% neutral-buffered formalin on days 7, 14, and 21 after the burn injury. After fixation, the tissues were trimmed, embedded, and divided into 5 mm thick sections and placed on glass slides (Immuno Coated slide, MUTO, Japan). These paraffin-embedded sections were treated with hematoxylin and eosin (H & E), Masson-trichrome (MT) stains, and immunohistochemistry (IHC) stain, as reported previously [7].

### 2.5. Inflammatory Cytokine Immunoassay

HMEC-1 cells were cultured, as described previously [11]. For the cell viability test, 1.0 × 10^5^ cells were seeded into a 24-well plate per well then treated with Rhubarb, Scutellaria root, Phellodendron bark, and Coptidis rhizome [12, 13]. The working concentrations of each herb material were 0.04, 0.12, 0.36, and 1.08 mg/mL. After incubation with HMEC-1 cells for 24 hours, each herb material's IC_50_ (half maximal inhibitory concentration) values were determined by counting of HMEC-1 number. Relative survival rates were shown as mean ± SD, taking the value of the control group as 100%. Through one-tailed test analysis, ∗ denotes statistical significance (*p* < 0.05) compared with control and represents two reproducible results.

Cells were treated with 0.1% DMSO, 0.8 mg/mL Rhubarb, 0.7 mg/mL Scutellaria root, 2.6 mg/mL Phellodendron bark, or 0.4 mg/mL Coptidis rhizome for 1 h and then untreated (DMSO and LPS groups) or treated with 0.2 g/mL LPS for an additional 24 h. The amount of human inflammatory cytokines in the cell suspension was determined using a human inflammatory cytokine multiplex ELISA kit (Arigo Biolaboratories, Hsinchu, Taiwan). All steps were performed as per the protocol provided by the manufacturer.

### 2.6. Reverse Transcription and Quantitative PCR

For HMEC-1, cells were pretreated with 0.5 mg/ml CA or 0.5 mg/ml TA for 2 hours and then treated with 0.2 g/ml LPS for another 24 hours. For the heat shock treatment of HaCaT, cells were incubated in a serum-free medium with 0.5 mg/ml CA or 0.5 mg/ml TA at 42°C for 15 min and then maintained at 37°C for another 48 hours. RNA was isolated from HMEC-1 and HaCaT cells using a purification kit (Protech Technology Enterprise, Taipei, Taiwan). Reverse transcription was performed using an RT kit (Protech Technology Enterprise, Taipei, Taiwan). Analysis of target gene expression by quantitative PCR was normalized with *β*-Actin. Primer sequences are listed in Table 4.

### 2.7. Cell Number Analysis

For HMEC-1 and RAW264.7, cells were pretreated with 0.5 mg/ml CA or 0.5 mg/ml TA for 2 hours and then treated with 0.2 *μ*g/ml LPS for another 24 hours. At the end of treatment, cells would be detached from culture plates using 0.25% trypsin EDTA solution (Thermo Fisher Scientific, Hualien, Taiwan) and then cell numbers could be counted.

## 3. Results and Discussion

### 3.1. Content of Reference Standards Present in Rhubarb, Scutellaria Root, Phellodendron Bark, and Coptidis Rhizome

**Figure 2:**
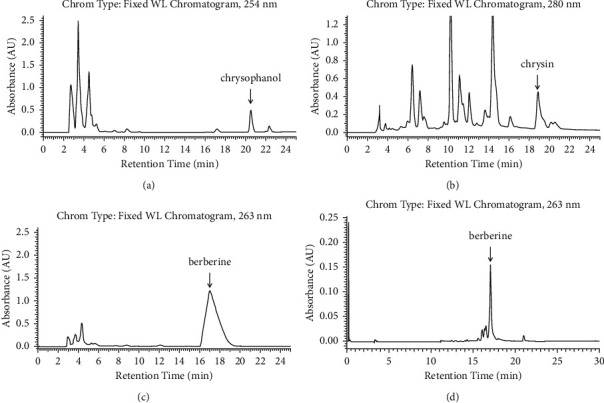
HPLC separation of reference compounds present in the extracts of herbal components. HPLC traces of (a) Chrysophanol in Rhubarb, (b) Chrysin in Scutellaria root, (c) Berberine chloride in Phellodendron bark, and (d) Berberine chloride in Coptidis rhizome.

One of the main problems associated with herbal medicine is the high batch-to-batch variability regarding the concentrations of its active components. The content of pure chemical reference standards in herbal products is therefore used as an indicator for quality control and standardization. To characterize the herbal materials, HPLC was used to determine the content of Chrysophanol in Rhubarb, Chrysin in Scutellaria root, and Berberine hydrochloride in Phellodendron bark and Coptidis rhizome. Accordingly, extraction yield of Rhubarb, Scutellaria root, Phellodendron bark, and Coptidis rhizome was 33.1%, 12.9%, 12.0%, and 10.0%, respectively. Total phenolic content (mg/g GAE) of Rhubarb, Scutellaria root, Phellodendron bark, and Coptidis rhizome was 29.4, 3.3, 10.0, and 76.4, respectively. The content of reference standards in Rhubarb, Scutellaria root, Phellodendron bark, and Coptidis rhizome was measured by HPLC (Figure 2). All calibration curves of reference compounds were linear over the concentration range studied (Table 5). A linear interpolation method was used to calculate the percentage by the mass of each reference standard in the analyzed herbal extracts.

### 3.2. Antibacterial Activity Assay

The MBC of herb materials used in this study against a variety of bacteria is shown in Table 6. *A. baumannii* Bouvet and Grimont (ATCC® 19606™), *A. baumannii* Bouvet and Grimont (ATCC® 17978™), *E. meningoseptica* BCRC 10677, *E. coli* DH5*α*, and *Pseudomonas aeruginosa* PAO1 are Gram-negative bacteria. In contrast, *P. acnes* PS023, *S. epidermidis* TCU-1 BCRC 81267, and *S. aureus* subsp. aureus TCU-2 BCRC 81268 are Gram-positive bacteria. The bactericidal effect of these four herbal extracts on Gram-positive bacteria is better compared to Gram-negative bacteria. The lowest MBC (^*∗*^) for each bacterial strain was distributed evenly among the four different herbs (Table 6). These results suggest that the combination of multiple herb materials could achieve the best bactericidal results.

During the clinical treatment, for example, one gram of “San Huang Powder” (at least 250 mg of each herb material powder) was used to spray over the wound area (12 cm^2^). If the thickness of the fluid that covers the wound area is 2 mm, the working concentration of each herbal powder on the site of the wound is approximately 104 mg/ml. Thus, considering the synergic effects from different herb materials, the active components in “San Huang Powder” must be sufficient to kill or inhibit bacterial growth on the wounds during treatment. Considering this calculation, it is clear why “San Huang Powder” has been directly applied onto the wound site without sterilization since ancient times. Although the antibacterial activities of Rhubarb, Scutellaria root, and Coptidis rhizome have been reported sporadically [14–17], we were the first to systematically compare them in this study. Moreover, it is common to detect multidrug-resistant *A. baumannii* in hospitalized patients [18], and the PS023 used in this study is an erythromycin- and clindamycin-resistant strain. Since the herbal extracts were effective against PS023 and two *A. baumannii* strains, the results also suggest that the herbal extract complex is promising in treating multidrug-resistant bacteria in the future.

### 3.3. Histopathological Evaluation of the Wound Healing Process

No animal was found dead or moribund during the study period. A partial-thickness burn was successfully produced on every animal with superficial second-degree severity, as confirmed by the formation of vesicles, epidermal discontinuity, superficial dermal necrosis, and inflammation.

**Figure 3:**
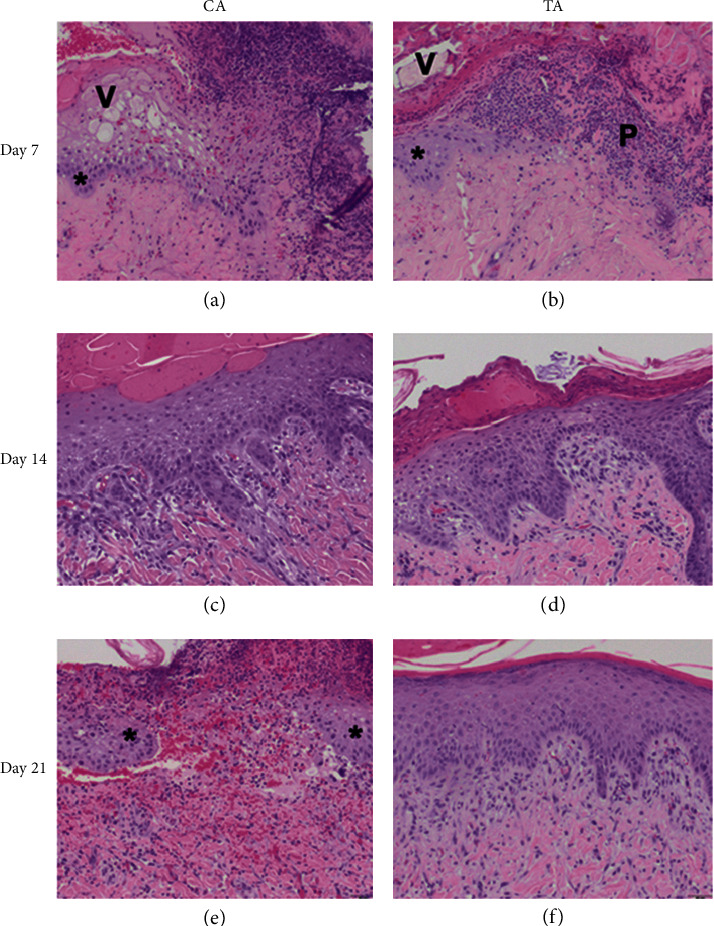
Results from H & E staining on different days after the application of herb, 100×. (a) CA on day 7, migrating epithelium (^*∗*^) and vesicle (*V*) formation with PMNL infiltration on wound surface. (b) TA on day 7, migrating epithelium (^*∗*^) and pustule (*P*) and vesicle (*V*) formation with PMNL infiltration on wound surface. (c) CA on day 14, bridging epithelium with fibroblasts and PMNL infiltration within wound area. (d) TA on day 14, bridging epithelium with fibroblasts and PMNL infiltration within wound area. (e) CA on day 28, migrating epithelium (^*∗*^) and an open wound between the edges. (f) TA on day 28, regenerated epithelium had sealed the wound without PMNL infiltration.

**Figure 4:**
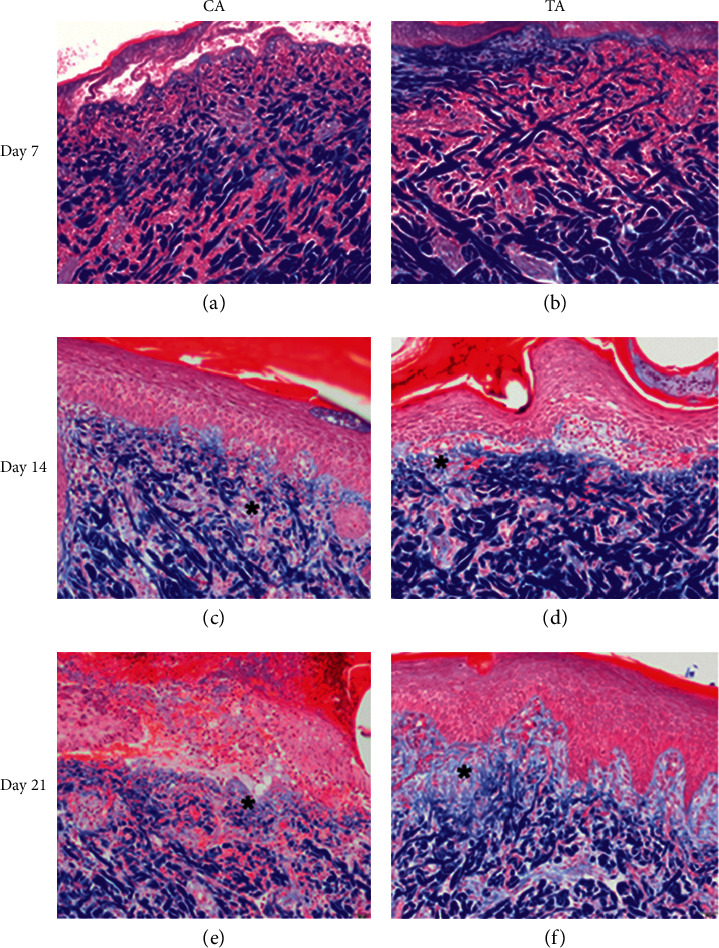
Results from Masson-trichrome staining on different days after the application of herb, 100×. (a) CA on day 7, no notable collagen deposition within wound area. (b) TA on day 7, no notable collagen deposition within wound area. (c) CA on day 14, the fibroblast had secreted minimal collagen in granulation tissue (^*∗*^). (d) TA on day 14, the fibroblast had secreted minimal collagen in granulation tissue (^*∗*^). (e) CA on day 28, unbridged epithelium with moderate collagen in granulation tissue (^*∗*^). (f) TA on day 28, bridged epithelium with moderate collagen in granulation tissue (^*∗*^).

**Figure 5:**
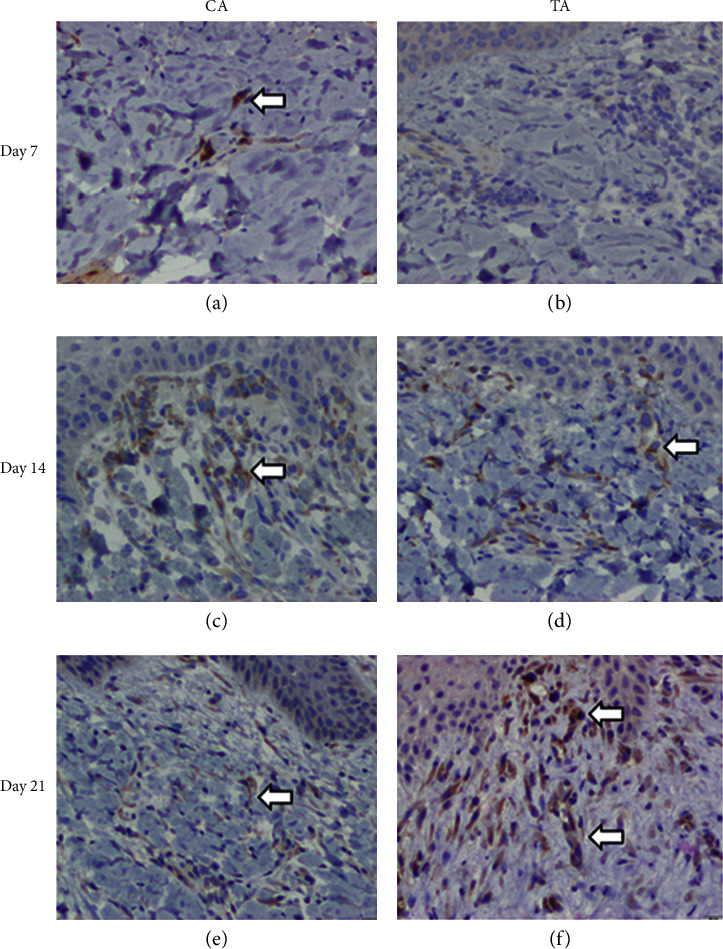
Results from IHC staining of VEGF on different days after the application of herb, 200×. (a) CA on day 7, weak VEGF signals from section. (b) TA on day 7, no positive VEGF result from section. (c) CA on day 14, weak VEGF signals from section. (d) TA on day 14, weak VEGF signals from section. (e) CA on day 21, weak VEGF signals from section. (f) TA on day 21, strong VEGF signals from section.

On day 7, epidermal basal cell migration and proliferation were observed under H&E staining, for all groups. As shown in Figures 3(a) and 3(b), the epidermal thickness was similar in both the CA and TA groups. During the early phase of wound healing, polymorphonuclear (PMNL) cell infiltration seemed more prominent in both groups. The scalding procedure not only induces localized tissue edema with transepidermal and superficial dermal necrosis but also causes vesicle and secondary pustule formation between the epidermal and dermal layers of skin. Due to the inflammatory stage of early wound healing, fibroblast proliferation and neo-formation of collagen matrix were not observed among all the groups on day 7, as can be seen in Figures 4(a) and 4(b). Results from the IHC staining of VEGF for angiogenesis revealed that, in the CA group, an increase in the expression of VEGF signals was seen compared to TA group (CA) (Figures 5(a) and 5(b)). This implied that the CA group had a better progression of wound healing at the early stage of acute burn injury.

The epidermal proliferation and thickness were prominent in both the CA and the TA groups on day 14 (Figures 3(c) and 3(d)). PMNL infiltration in the TA group was significantly less than that observed in the CA group, which means the wound healing step of the CA group is still in an inflammatory state. However, the MT staining showed that the collagen bundles between the dermal layers were more prominent in the TA group than in the CA group. Therefore, the proliferation and regeneration steps had started in the TA group, where the new tissue was rebuilt with collagen and extracellular matrix (Figures 4(c) and 4(d)). Hence, at this point of observation, the healing rate at the proliferation stage of acute burn injury was as follows: TA > CA.

On day 21, both groups had complete epidermal regeneration without significant differences under H & E staining (Figures 3(e) and 3(f)). MT staining also revealed that the collagen bundles over the epidermal-dermal junction and upper dermis were more thickened and compact in the experimental groups compared to the CA group (Figures 4(e) and 4(f)). Moreover, the PMNL infiltration was absent in TA, whereas PMNL remained in the CA group (Figures 3(e) and 3(f)). The newly formed blood vessels can provide oxygen and nutrients to promote wound repair. Since the VEGF signal in the TA group was higher than that in the CA group, angiogenesis can help the tissue heal faster in the TA group than in the CA group (Figure 5). Therefore, the healing rate of acute burn wounds at this stage was as follows: TA > CA.

During the process of wound healing, the pigs felt itchy and would actively rub the wound or even try to remove the dressing. The blisters on the burn wound site were quite fragile. Because the particle size of herb materials is enormous and coarse, the friction from the herb particles led to rough wound surfaces in all experimental groups. Furthermore, the dark-colored herb materials were mixed with the exudate to form black scabs on days 14 and 21. The scab made it challenging to observe the healing process of the wound based on appearance (Figure S1). The wounds were superficial second-degree burns, and granulation tissues were observed on day 14 (Figure S1). Therefore, it is less likely to heal with significant scarring and wound contracture [19].

In short, a second-degree superficial burn was created successfully in each pig, as evidenced by epidermal and upper dermal necrosis, vesicle formation, and inflammation. In the experimental groups, an early induction of epidermal basal cell migration and dermal endothelial cell angiogenesis was observed, along with the activation of immune response with PMNL infiltration for foreign pathogens on day 7, during the initial inflammatory stage of wound healing. On day 14 of wound healing (proliferation stage), the TA group had more prominent epidermal regeneration and increased dermal fibroblast proliferation and collagen synthesis. Finally, on day 21 of the wound healing process (proliferation and remodeling stage), a significant increase in fibroblast activities, collagen synthesis, and angiogenesis was observed in the TA group, which helped in an improved healing rate of the acute burn wound. The main difference in the composition between the CA and TA groups was the presence of Rhubarb in the latter groups.

### 3.4. Inflammatory Cytokine Immunoassay

**Figure 6:**
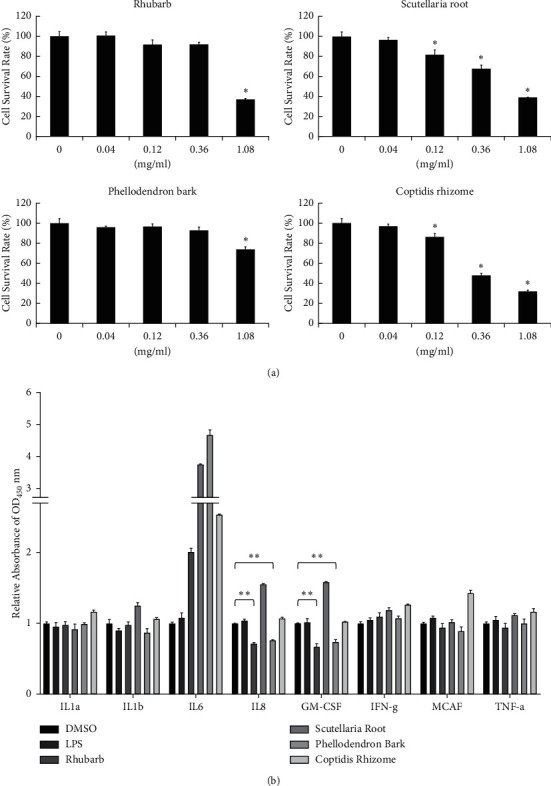
The *in vitro* assay of the anti-inflammatory activity of herb materials used in this study. (a) HMEC-1 cells were treated with 0.04, 0.12, 0.36, and 1.08 mg/mL herb, including Rhubarb, Scutellaria root, Phellodendron bark, and Coptidis rhizome for 24 h. Cell numbers were calculated and shown as mean ± SD. The IC_50_ (half maximal inhibitory concentration) values of Rhubarb, Scutellaria root, Phellodendron bark, and Coptidis rhizome for HMEC-1 cell were 0.88 ± 0.05, 0.71 ± 0.06, 2.65 ± 0.59, and 0.44 ± 0.05 mg/mL, respectively. The 0 group is without herb treatment, only DMSO solvent as control. The cell survival rate of control was taken as 100%. (b) HMEC-1 cells were treated with 0.1% DMSO, 0.8 mg/mL Rhubarb, 0.7 mg/mL Scutellaria root, 2.6 mg/mL Phellodendron bark, or 0.4 mg/mL Coptidis rhizome for 1 h and then untreated (DMSO and LPS groups) or treated with 0.2 g/mL LPS for additional 24 h. Relative folds of OD450 nm values were calculated and shown as mean ± SD, taking the value of DMSO group as 1.0. Through one-tailed test analysis, ^*∗∗*^denotes statistical significance (*p* < 0.005) compared with DMSO and represents two reproducible results.

The toxicity of each herb material was first determined by MTT assay [20]. However, the deep color of the herb extract led to significant errors. Therefore, their IC_50_ (half maximal inhibitory concentration) values for HMEC-1 cells were determined by counting cell numbers directly (Figure 6(a)). The *in vitro* anti-inflammatory activities of the four herb materials were then measured. Because the solubility and cytotoxicity of each herb material in DMSO are different, the maximum possible dose for each component was used for this assay, *i.e.*, 0.8 mg/mL Rhubarb, 0.7 mg/mL Scutellaria root, 2.6 mg/mL Phellodendron bark, and 0.4 mg/mL Coptidis rhizome.

Previous reports have indicated that the concentrations of more than ten different inflammatory cytokines, including interleukin-6 (IL-6), interleukin-8 (IL-8), and granulocyte-macrophage colony-stimulating factor (GM-CSF), were significantly higher in the serum of a burn patient than in controls [21]. As shown in Figure 6(b), the treatment with Rhubarb and Phellodendron bark led to a decrease in the levels of inflammatory cytokines, IL-8, and GM-CSF on LPS-induced HMEC-1 cells.

The results obtained from the histopathologic evaluation of the tissues suggested that, on day 21, CA had a slower healing rate. The main difference between CA and TA groups was the presence of Rhubarb. These results were in line with a previous report and suggested that Rhubarb may play a vital role in burn wound healing [4].

### 3.5. Reverse Transcription and Quantitative PCR

**Figure 7:**
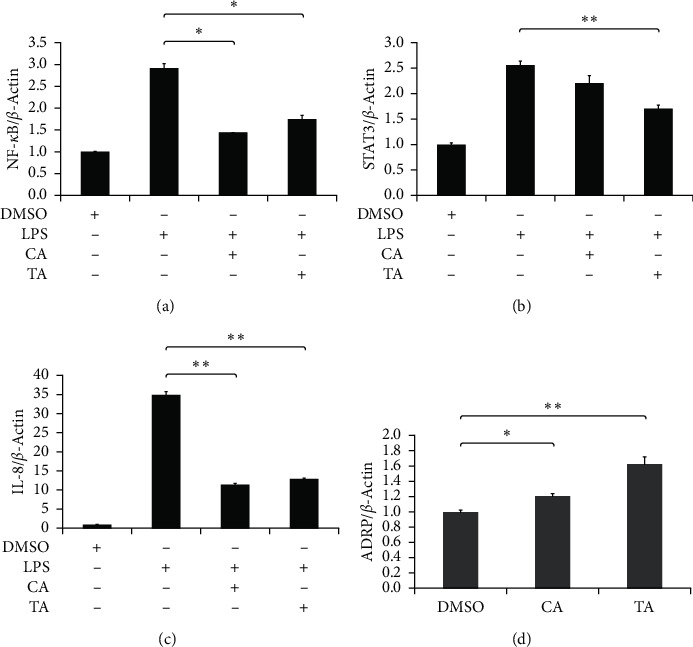
The effects of the inclusion of Rhubarb on inflammatory and lipogenesis-related genes. HMEC-1 cells were pretreated with 0.5 mg/ml CA or TA for 2 h then with 200 ng/ml LPS (a, b). HaCaT were treated with 0.5 mg/ml CA or TA from heat shock for 15 min to incubate for 48 h (c, d). Cellular RNA was isolated and then mRNA expression of (a) NF-*κ*B, (b) STAT3, (c) IL-8, and (d) ADRP were determined by reverse transcription quantitative PCR. *β*-actin was used as internal control. ^*∗*^*p* < 0.05and ^*∗∗*^*p* < 0.005 present statistical significance compared to CA group.

To further investigate the gene expression difference in the presence or absence of Rhubarb in San Huang Powder during the wound healing process, RT and qPCR experiments were carried out. LPS was first used to induce inflammatory conditions in human endothelial cells, HMEC-1. NF-*κ*B [22–25] and STAT3 [26–29] are proinflammatory factors to form a transcriptional complex which regulates the inflammatory response related to IL-8 gene expression [30–35]. As shown in Figures 7(a)–7(c), the gene expression of NF-*κ*B, STAT3, and IL-8 was significantly inhibited after the treatment of LPS in both CA and TA groups. The decrease of LPS-induced NF-*κ*B and IL-8 expression in the CA group is slightly higher than that in the TA group; however, the reduction of LPS-induced STAT3 expression in the TA group is more elevated than in the CA group. Therefore, there was no significant difference in the anti-inflammatory response between the CA and TA groups.

A previous study has shown that knockdown of the gene encoding adipose differentiation-related protein (ADRP) could impair wound healing in mice [36]. Other studies also indicated that lipid signaling molecules could regulate the wound healing process [37–42]. Moreover, lipids might play a role in the proliferation and migration of fibroblasts [43–48]. The heat-shocked keratinocyte model was used to mimic a burn wound. As shown in Figure 7(d), the treatment of CA and TA could lead to an increase of ADRP expression by 21% and 68%, respectively, in heat-shocked keratinocytes compared with a solvent control (DMSO). ADRP expression is associated with lipid storage as a marker of lipid accumulation in cells. The upregulation of ADRP in both groups might play a role in the wound healing process in this study. The inclusion of Rhubarb in San Huang Powder seems to help wound healing in this respect.

**Figure 8:**
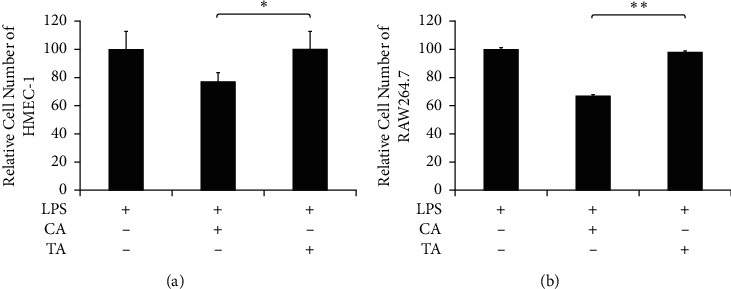
The effects of the inclusion of Rhubarb on cell growth. (a) HMEC-1 and (b) RAW264.7 cells were pretreated with 0.5 mg/ml CA or TA for 2 h and then with 0.2 *μ*g/ml LPS for 24 h. Relative cell number were calculated and LPS group refer to 100. Through one-tailed test analysis, ^*∗*^ and ^*∗∗*^ present statistical significance (*p* < 0.05 and *p* < 0.005, resp.) as denoted.

The cell numbers of HMEC-1 and mouse macrophages (RAW264.7) were also measured after LPS and herb extract treatment. As shown in Figures 8(a) and 8(b), the cell number for the CA group decreased by 26% for HMEC-1 and 33% for RAW264.7, respectively, compared to the group treated with LPS only. On the other hand, the cell number for the TA group was still close to the groups treated with LPS only. In other words, treatment with the Phellodendron bark, Scutellaria root, and Coptidis extract mixture led to the reduction of endothelium cell and macrophage *in vitr*o [37–40]. The inclusion of Rhubarb in San Huang Powder seems to reverse this effect, although the mechanism remains unclear. Previous studies have shown that Rhubarb extract played a protective role against radiation-induced brain injury and neuronal cell apoptosis by inhibiting ROS (Reactive Oxygen Species) formation [41]. The endothelial dysfunction and tissue injury caused by oxidative stress at the inflammatory site have been well documented [42–47]. Therefore, this might account for the efficacy for the TA group being better than that for the CA group on histopathological evaluation.

## 4. Conclusions

Our results provide a basis to understand why “San Huang Powder” without sterilization can be clinically used to treat wounds directly since ancient times. This study also shows the advantages of using multiple herb materials simultaneously on the wound sites to control infection during treatment. Moreover, the herbal extract complex sheds some light on treating multidrug-resistant bacteria in the future.

Both groups possessed similar *in vitro* anti-inflammatory activity. However, the exclusion of Rhubarb resulted in a decrease of endothelium and macrophage cell numbers under an inflammatory state. Therefore, the inclusion of Rhubarb was recommended for the recipe of “San Huang Powder” for healing efficacy of burn wounds. The results obtained in this study also provide the basis to improve the preparation of this traditional medicine. The next generation of this herbal product is probably in the form of sterile burn wound cream.

## Figures and Tables

**Table 1:** The recipes of two different types of “San Huang Powder” used in this study.

Group	Ingredient
Control (CA)	Scutellaria root : Phellodendron bark : Coptidis rhizome = 1 : 1 : 1
Test article (TA)	Rhubarb : Scutellaria root : Phellodendron bark : Coptidis rhizome = 1 : 1 : 1 : 1

**Table 2:** HPLC elution programs for Rhubarb, Scutellaria root, Phellodendron bark, and Coptidis rhizome.

Rhubarb		Scutellaria root		Phellodendron bark		Coptidis rhizome	
Time (min)	Eluent (B%)	Time (min)	Eluent (B%)	Time (min)	Eluent (B%)	Time (min)	Eluent (B%)
0–10	35	0–2	15	0–5	20	0–5	0
10–25	35–100	2–6	15–30	5–25	20–30	5–16	0–80
25–30	100	6–20	30–40	25–35	30–100	16–21	80–100
		20–22	40–50	35–40	100	21–30	100

**Table 3:** Treatment groups and duration.

Group	Treatment	Study period (biopsy samples taken)		
		7 days	14 days	21 days
CA	1 g/day	3	3	3
TA	1 g/day	3	3	3

**Table 4:** Primer sequences for RT-qPCR of target genes in this study.

Target gene	Primer sequence
ADRP	F: GGCTAGACAGGATTGAGGAGAG
	R: TCACTGCCCCTTTGGTCTTG
IL-8	F: CTCTCTTGGCAGCCTTCCTGA
	R: CCCTCTGCACCCAGTTTTCCTT
NF-*κ*B	F: CCTGGATGACTCTTGGGAAA
	R: TCAGCCAGCTGTTTCATGTC
STAT3	F: CATATGCGGCCAGCAAAGAA
	R: ATACCTGCTCTGAAGAAACT

**Table 5:** HPLC calibration curves of reference compounds, including regression equations, coefficients of determination (*R*^2^), and calibration ranges.

Reference compound	Regression equation	*R* ^2^	Calibration range	Mass percentage (%)
Chrysophanol in Rhubarb	*y* = 37412*x* + 8906.3	0.999	0.15–5 *μ*g	0.47
Chrysin in Scutellaria root	*y* = 1951500*x* − 96365	0.998	0.1–10 *μ*g	0.37
Berberine chloride in Phellodendron bark	*y* = 48857*x* + 672075	0.999	1.25–40 *μ*g	1.06
Berberine chloride in Coptidis rhizome	*y* = 42135*x* + 151920	0.996	0.5–10 *μ*g	2.14

**Table 6:** The minimal bactericidal concentration (MBC) of the herbal materials used in this study.

Bacterial strains	Rhubarb (mg/ml)	Scutellaria root (mg/ml)	Phellodendron bark (mg/ml)	Coptidis rhizome (mg/ml)
*A. baumannii* Bouvet and Grimont ATCC 19606	15.6^*∗*^	31.3	125	31.3
*A. baumannii* Bouvet and Grimont ATCC 17978	15.6^*∗*^	62.5	>250	125
*E. meningoseptica* BCRC 10677	<7.8^*∗*^	15.6	125	31.3
*E. coli* DH5*α*	125	31.3^*∗*^	>50	31.3^*∗*^
*P. acnes* PS023	15.6	31.3	7.8^*∗*^	15.6
*P. aeruginosa* PAO1	15.6	7.8	15.6	15.6
*S. epidermidis* TCU-1 BCRC 81267	<7.8^*∗*^	15.6	<7.8^*∗*^	<7.8^*∗*^
*S. aureus* subsp. aureus TCU-2 BCRC 81268	31.3	31.3	15.6	<7.8^*∗*^

^*∗*^The lowest concentration to completely kill the specific strain.

## Data Availability

The data obtained in this research can be made available from the corresponding author upon request or from the indicated sources.

## Supplementary Materials

Supplementary Materials Figure S1: representative photomicrographs showing the time course of wound healing on days 0, 7, 14, and 21.
