# Supplementary material for: Antimicrobial and Immunomodulatory Activity of Herb Extracts Used in Burn Wound Healing: “San Huang Powder”
Source: Evid Based Complement Alternat Med. 2021 Oct 12;2021:2900060. doi: 10.1155/2021/2900060 (PMC8526243; doi:10.1155/2021/2900060)
Supplement: Supplementary Materials — Figure S1: representative photomicrographs showing the time course of wound healing on days 0, 7, 14, and 21. [file 2900060.f1.pdf]

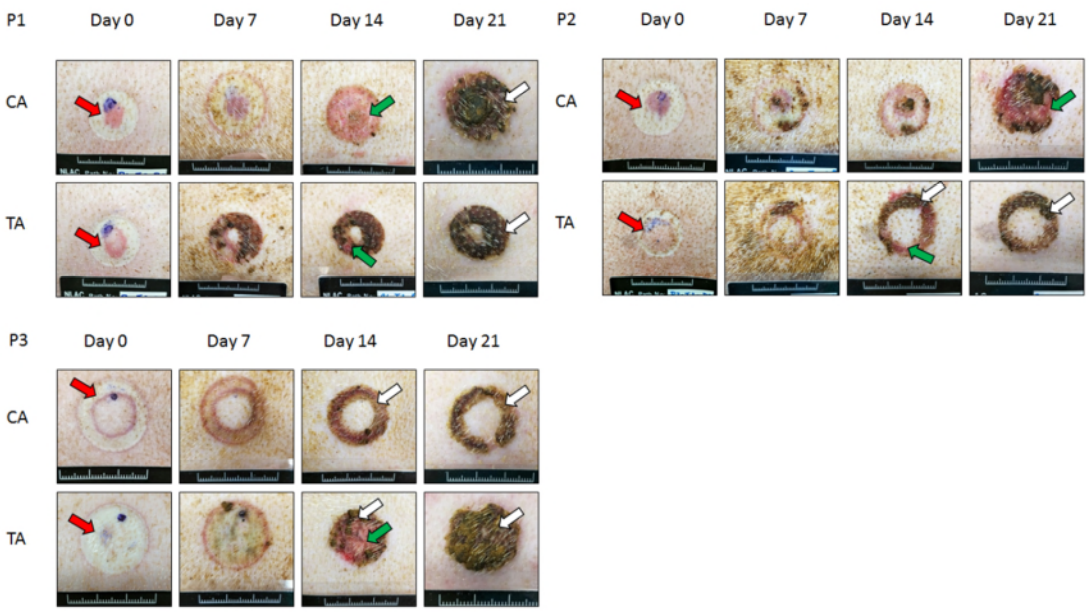

Figure S1. Representative photomicrographs showing the time course of wound healing on days 0, 7, 14, and 21. Red arrow: ischemic necrosis; green arrow: new pinkish granulation tissue; white arrow: crusted skin mixed with exudate and herbal materials.
